# Postprandial Hypotension in Adults: Exploring Insulin Dynamics During a Mixed Meal Test

**DOI:** 10.3390/nu17030479

**Published:** 2025-01-28

**Authors:** Rahime Evra Karakaya, Abbas Ali Tam, Sevgül Fakı, Gülsüm Karaahmetli, Didem Özdemir, Reyhan Ersoy, Oya Topaloğlu

**Affiliations:** 1 Department of Nutrition and Dietetics, Faculty of Health Sciences, Ankara Yıldırım Beyazıt University, 06760 Ankara, Türkiye; 2 Department of Endocrinology and Metabolism, Faculty of Medicine, Ankara Yıldırım Beyazıt University, 06800 Ankara, Türkiye; aatam@aybu.edu.tr (A.A.T.); dozdemir@aybu.edu.tr (D.Ö.); reyhanersoy@aybu.edu.tr (R.E.); otopaloglu@ybu.edu.tr (O.T.); 3 Department of Endocrinology and Metabolism, Ankara Bilkent City Hospital, 06800 Ankara, Türkiye; black_snowtr@yahoo.com (S.F.); gulsumgedik85@gmail.com (G.K.)

**Keywords:** hypotension, hypoglycemia, insulin, meal, adults, postprandial

## Abstract

**Background/Objectives:** Postprandial hypotension (PPH) is an important clinical condition in patients presenting with postprandial symptoms. The aims of this study were to determine the prevalence of PPH in patients with postprandial symptoms and to investigate the relationship between PPH and insulin, particularly in healthy adults. **Methods**: This study was conducted with 111 adult patients who were admitted to the clinic due to postprandial symptoms. Patients underwent the mixed meal test (MMT). Blood glucose, insulin, and C-peptide levels were measured at 0, 30, 60, 90, 120, 180, 240, and 300 min along with systolic blood pressure (sBP), diastolic blood pressure (dBP), and heart rate measurements during the MMT. **Results:** Serum adrenocorticotropic hormone (ACTH) levels were similar (*p* > 0.05), and cortisol levels were found to be higher in individuals without PPH compared to those with PPH before the MMT (*p* = 0.014). During the MMT, 23 patients (23.2%) had PPH. At the beginning of the test, serum glucose, insulin, C-peptide, and heart rate values were similar in patients with and without PPH; however sBP and dBP were significantly higher in the PPH group (*p* = 0.002 and *p* = 0.010, respectively). No correlation was found between sBP and insulin, glucose, and C-peptide at any time during the MMT except for a moderately significant positive correlation between glucose and sBP at 90 min in patients with PPH (r = 0.490, *p* = 0.018). A moderately negative correlation was found between the magnitude of sBP fall between 30 and 60 min and insulin and C-peptide levels in people with PPH (r = −0.420, *p* = 0.046; r = −0.564, *p* = 0.005; respectively). However, no significant relationships were observed between the magnitude of sBP fall at other time points and blood parameters (*p* > 0.05). **Conclusions:** A significant portion of adults with postprandial symptoms might have PPH, contributing to these symptoms. The lack of a relationship between insulin and glucose suggests that other physiological mechanisms beyond insulin and glucose may play a role in the pathogenesis of PPH in healthy individuals. Therefore, further research is needed to better understand the underlying causes of PPH.

## 1. Introduction

Postprandial hypotension (PPH) is defined as a reduction in sBP by 20 mmHg or more within two hours after a meal, or a decrease in sBP to 90 mmHg or lower after a meal when the pre-meal sBP is greater than 100 mmHg [1]. It is commonly observed in the geriatric population, as well as in individuals with type 2 diabetes mellitus (DM), Parkinson’s disease, and spinal cord injuries [2,3,4,5]. Risk factors generally include certain medications, meal type and timing, pre-meal blood pressure, and specific comorbidity conditions [6]. Despite its low awareness, even asymptomatic PPH can lead to increased risks of falls, syncope, cerebrovascular disease, angina, and cardiovascular mortality [1,7,8,9].

Idiopathic postprandial hypoglycemia (IPH), on the other hand, is a condition characterized by autonomic and neuroglycopenic symptoms accompanied by low plasma glucose levels occurring 2 to 5 h after food intake [10]. Although PPH and idiopathic postprandial hypoglycemia are distinct conditions, both are postprandial phenomena that may share overlapping mechanisms, including exaggerated insulin response and impaired autonomic regulation [11]. The mechanisms thought to contribute to idiopathic postprandial hypoglycemia include high insulin sensitivity, insulin resistance, an exaggerated insulin response associated with increased glucagon-like peptide (GLP)-1, renal glycosuria, and impaired glucagon response. These mechanisms may also exacerbate the splanchnic blood pooling and vasodilation observed in PPH [12]. Studies on patients with suspected idiopathic postprandial hypoglycemia are quite limited, with many being older studies involving small sample sizes. These studies report that a significant proportion of patients with hypoglycemic symptoms did not show biochemical hypoglycemia on an oral glucose tolerance test (OGTT) or MMT [13,14,15,16].

Although the pathophysiology of PPH is not fully understood, the most commonly proposed mechanisms include inadequate sympathetic nervous system response to splanchnic blood pooling after a meal, impairments in baroreflex function, insulin-induced vasodilation, the release of vasodilatory gastrointestinal peptides, and rate of gastric emptying [1,17]. Despite these proposed mechanisms, the exact role of insulin in the development of PPH remains unclear [18]. A study found that insulin levels were similar in response to both oral and intravenous glucose, even though a fall in blood pressure occurred only after oral glucose [19]. Additionally, PPH has also been observed in individuals with type 1 diabetes, who are, by definition, insulin dependent [20]. These findings indicate that insulin may play an inconsistent and limited role as a mediator of PPH. In another study including older adults [11], it was found that those with PPH had a greater postprandial increase in blood glucose and insulin levels compared to those without PPH, and the maximum drop in postprandial sBP was associated with the maximum increase in insulin but not with the maximum increase in blood glucose. This overlap suggests that insulin and glucose dynamics, a common feature in idiopathic postprandial hypoglycemia, may play a role in PPH as well. However, studies investigating the relationship between PPH and insulin and glucose levels are limited. Additionally, although PPH is more commonly observed in older adults, it is important to consider PPH in younger adults who present with symptoms of postprandial hypoglycemia. This study was conducted to determine the prevalence of PPH in adults with postprandial symptoms and to investigate the relationship between PPH and insulin and glucose levels.

## 2. Materials and Methods

### 2.1. Study Protocol

The study was conducted prospectively between April and September 2024 with patients who underwent an MMT at the Endocrinology Clinic of Ankara Bilkent City Hospital. During the MMT protocol, glucose, insulin, and C-peptide measurements were taken at specified intervals over a 5 h period, along with blood pressure and heart rate measurements. Symptoms, if present, were also recorded through patient inquiry. A total of 117 individuals participated in the study; however, after excluding those with incomplete blood test results or missing blood pressure measurements throughout the study period, the final analysis was conducted with 111 participants.

### 2.2. Participants

Patients aged 18–65 years who were suspected to have reactive hypoglycemia due to their symptoms and required confirmation of the diagnosis were included, and a mixed meal test was scheduled. Based on patient complaints at the time of clinic admission, 90 patients presented with adrenergic symptoms, 38 with neuroglycopenic symptoms, and 17 with both adrenergic and neuroglycopenic symptoms. Adrenergic symptoms were defined as palpitation, sweating, tremor, anxiety, hunger, nervousness, irritability, nausea, diaphoresis, and paresthesias, while neuroglycopenic symptoms included headache, fatigue, dizziness, confusion, behavioral changes, visual disturbances, seizure, fainting, and coma. Also, patients without tachycardia on resting electrocardiogram (ECG) were included in the study.

Individuals with prediabetes or DM, those aged 65 and above, patients using antihypertensive medications or with known hypertension, those with active infections, congestive heart failure, peripheral artery disease, or advanced-stage dementia, and individuals who could not provide a history or were unable to cooperate were excluded from the study. Additionally, patients using medications that could induce hypoglycemia (oral antidiabetics, insulin, beta-blockers, hydroxychloroquine, bromocriptine, etc.) or those with conditions that could cause hypoglycemia (uncontrolled hypothyroidism, adrenal insufficiency, growth hormone deficiency, etc.), as well as those taking medications affecting gastric emptying (such as GLP-1 analogs) or nutrient absorption (such as orlistat) were excluded from the study.

### 2.3. Mixed Meal Test (MMT)

The patients were put on an isocaloric diet containing at least 150 gr/day carbohydrate for at least 3 consecutive days before the test. Smoking and exercise were avoided 24 h prior to the test. The test was started between 8.00 and 9.00 am after a 10–12 h fast. After insertion of a venous canula, blood was taken for baseline plasma glucose, insulin, and C-peptide measurements, and blood pressure and heart rate were measured at time 0. Then, patients consumed a breakfast of 9 kcal/kg bodyweight containing 60% carbohydrate, 20% fat, and 20% protein prepared by the dietician. The meal included skimmed milk, white cheese, whole wheat bread, an apple, and sugar. After ingestion, serial blood samples were taken for the same parameters at 30, 60, 90, 120, 180, 240, and 300 min. Also, blood pressure and heart rate measurements were performed at these periods. Blood sampling and measurements were performed while the patients were seated. Between measurements, the patients mostly remained seated or moved around within the room. Long-distance walking, exercise, or smoking was not permitted during the test. The blood samples were sent to the laboratory immediately after each sampling.

Also, the symptoms were formally assessed by nurses/healthcare staff during the test using a form containing a list of adrenergic and neuroglycopenic symptoms to inquire about them.

### 2.4. Laboratory Parameters

Before the MMT, hemoglobin A1c (HbA1c), fasting and postprandial glucose, fasting and postprandial insulin, fasting C-peptide, adrenocorticotropic hormone (ACTH), and cortisol levels were recorded after an 8 h fast on the clinic admission day. During the MMT, serum glucose, insulin, and C-peptide levels were measured at 0, 30, 60, 90, 120, 180, 240, and 300 min. T0 blood samples and measurements were taken before meal ingestion on the test day, and participants were instructed to consume the meal within 15 min. Glucose measurements were performed using the hexokinase method and the Atellica analyzer with a reference range of 70–99 mg/dL, coefficient of variation (CV) ranging from 1.3% to 1.9% for low and high control levels, and a Westgard-recommended CV of 2.8% (Siemens Healthcare Diagnostics, Deerfield, IL, USA). Insulin and C-peptide levels were measured using chemiluminescent immunoassay methods with the Atellica analyzer and assays. For insulin, the reference range was 3–25 mU/L, CV ranged from 1.7% to 2.4%, and the Westgard-recommended CV was 10.6%. For C-peptide, the reference range was 0.81–3.85 µg/L, CV ranged from 5.6% to 7.4%, and the Westgard-recommended CV was 8.6%.

### 2.5. Blood Pressure and Heart Rate

Blood pressure and heart rate measurements were performed using an automatic measurement device during blood sampling times. These measurements were performed after patients had rested for at least 15 min. During resting time, other preparatory tasks were carried out, such as preparing blood collection tubes and meal preparations. A stabilization period of 15 min was provided to ensure that the measurements were accurate and not influenced by initial anxiety or activity. The first measurements were performed twice, and subsequent measurements were performed once. While the patient was seated, the cuff was inflated, and simultaneous measurements of the arterial pressure and heart rate in the forearm were performed using the Omron M4 automatic upper arm blood pressure monitor. Blood pressure and heart rate measurements were performed immediately before blood collection.

### 2.6. PPH Diagnostic Criteria

PPH was diagnosed when sBP decreased by ≥20 mmHg or sBP decreased to below 90 mmHg from a pressure of ≥100 mmHg during the MMT.

### 2.7. Postprandial Hypoglycemia Diagnostic Criteria

Patients with a blood glucose level of <55 mg/dL at any time (excluding the 0th min) during the MMT were defined as having postprandial hypoglycemia.

### 2.8. Statistical Analyses

Statistical analyses were performed using SPSS v.26 software. The Shapiro–Wilk test was used to assess the normality of the data. Continuous variables were expressed as median and range [IQR]. To compare continuous variables between groups, an independent samples *t*-test was used for data that followed a normal distribution, and the Mann–Whitney U test was used for non-normally distributed data. Categorical variables were expressed as counts and percentages (*n* (%)), and the chi-square test or Fisher’s exact test (when expected frequencies were less than 5) was used to compare categorical variables between groups. The maximum rise in heart rate and the maximum fall in sBP compared to baseline (0 min) were identified at their respective time points up to 300 min. The magnitude of the fall in sBP was calculated as the delta (Δ) between each consecutive time point, comparing the changes from baseline (0 min) to the following time points: Δ(0–30 min), Δ(30–60 min), Δ(60–90 min), Δ(90–120 min), Δ(120–180 min), Δ(180–240 min), and Δ(240–300 min). Pearson correlations were used to evaluate the relationships between variables with normal distribution, while Spearman correlation analysis was used for non-normally distributed variables. The correlation coefficient was classified as follows: <0.2 as very weak, 0.2–0.4 as weak, 0.4–0.6 as moderate, 0.6–0.8 as strong, and >0.8 as very strong. A *p*-value of <0.05 was considered statistically significant in all analyses.

## 3. Results

### 3.1. Demographic and Clinical Characteristics

The data of 111 patients were analyzed. There were 86 (77.5%) female and 25 (22.5%) male patients, and the mean age was 42 [17] years. Adrenergic symptoms were present in 90 (81.1%) patients and neuroglycopenic symptoms were present in 38 (34.2%) patients. The laboratory parameters are given in Table 1. Postprandial hypoglycemia was detected in 12 (10.8%) patients and PPH was observed in 26 (23.4%) patients during the MMT (Table 1).

### 3.2. Laboratory Findings During the MMT

**Figure 1:**
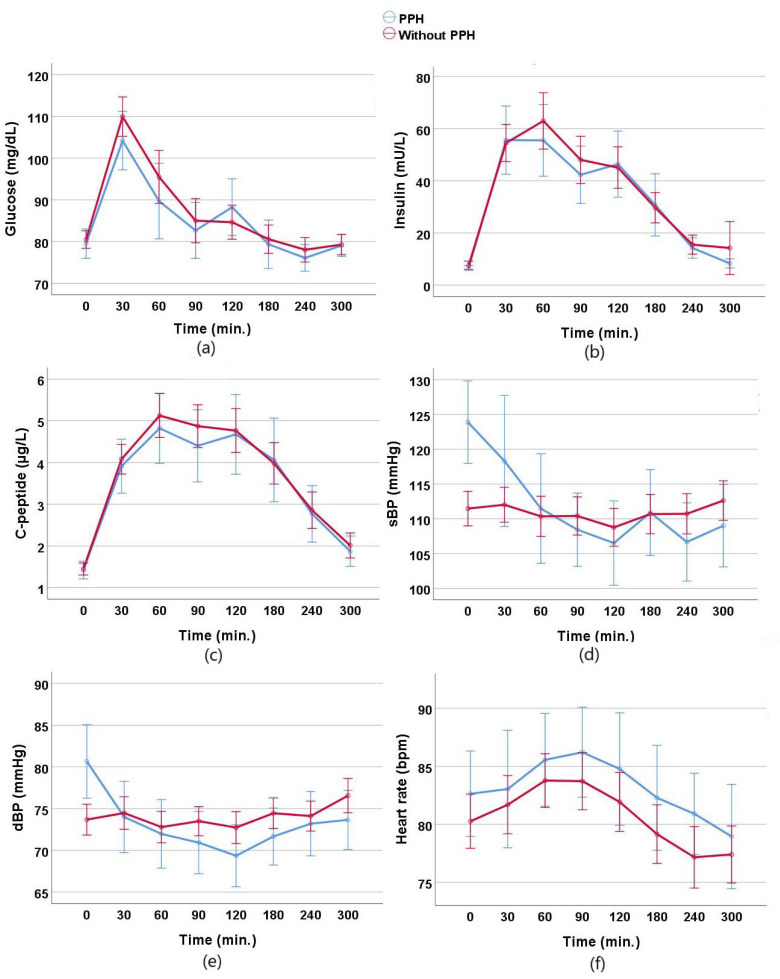
Changes in laboratory findings, blood pressure, and heart rate measurements during the mixed meal test (MMT) in individuals with and without PPH: (**a**) changes in glucose levels, (**b**) changes in insulin levels, (**c**) changes in C-peptide levels, (**d**) changes in systolic blood pressure (sBP) levels, (**e**) changes in diastolic blood pressure (dBP) levels, and (**f**) changes in heart rate levels.

Changes in glucose, insulin, C-peptide, sBP, dBP, and heart rate during the MMT in individuals with and without PPH are shown in Figure 1.

### 3.3. Comparison of Patients with and Without PPH During MMT

After excluding patients with postprandial hypoglycemia (*n* = 12) during the MMT, the demographic and laboratory findings of patients with and without PPH were compared. There were 23 (23.2%) patients with and 76 patients (76.8%) without PPH. Age, sex distribution, and presence of adrenergic or neuroglycopenic symptoms were similar between groups at the time of admission. However, basal cortisol levels were higher in patients without PPH (*p* = 0.014). Baseline glucose, insulin, and C-peptide levels on the MMT day were similar in the two groups. Patients with PPH had higher basal sBP and dBP compared to patients without PPH (*p* = 0.002 and *p* = 0.010, respectively). There was no significant difference in the lowest glucose, highest insulin, and highest C-peptide during the MMT in the two groups. The maximum fall in sBP was higher in the PPH group (*p* < 0.001), and the maximum rise in heart rate was similar between groups (*p* = 0.606) (Table 2).

### 3.4. Correlations Between Blood Pressure and Other Parameters

After excluding patients with postprandial hypoglycemia, there were moderately positive relationships between sBP and glucose at 60 min and 90 min (r = 0.419, *p* < 0.001; r = 0.407, *p* < 0.001, respectively). There were also weakly positive relationships between sBP and insulin at 60 min and 90 min (r = 0.298, *p* = 0.003; r = 0.344, *p* < 0.001, respectively). Weakly positive relationships were also found between sBP and C-peptide at 60 min and 90 min (r = 0.263, *p* = 0.008; r = 0.373, *p* < 0.001, respectively) (Table 3).

After excluding patients with postprandial hypoglycemia, correlations between sBP with insulin, glucose, and C-peptide in patients with PPH were examined. No correlations were found, except a moderately significant positive correlation between glucose and sBP at 90 min (r = 0.490, *p* = 0.018) (Table 4).

Weakly negative correlations were found between the magnitude of sBP fall between 30 and 60 min and glucose, insulin, and C-peptide levels in all individuals (r = −0.312, *p* = 0.002; r = −0.241, *p* = 0.016; r=-0.221, p=0.028; respectively) (Table 5).

Moderately negative correlations were found between the magnitude of sBP fall between 30 and 60 min and insulin and C-peptide levels in people with PPH (r = −0.420, *p* = 0.046; r = −0.564, *p* = 0.005; respectively) (Table 6).

## 4. Discussion

In the present study, we showed that 23.2% of adults who exhibited postprandial symptoms and underwent an MMT due to suspected postprandial hypoglycemia had PPH. PPH is a clinical condition typically seen in older adults and often goes underdiagnosed. Studies on the prevalence of PPH have mainly focused on older adults and individuals with additional risk factors, such as Parkinson’s disease and type 2 DM. In a systematic review of 20 studies, PPH prevalence was reported to be 24–38% in healthy older adults and nursing home residents, 20–91% in hospitalized geriatric patients, 40% in type 2 DM patients, and 40–100% in Parkinson’s disease patients [21]. A recent meta-analysis found that 40.5% of older adults had PPH [22]. Studies examining PPH prevalence in younger adults are quite limited. In one study involving both young and older adults, the prevalence of PPH was 25% in normotensive young adults, 29% in hypertensive young adults, 43% in normotensive older adults, and 63% in hypertensive older adults [23]. In a study comparing adults with and without obesity aged 18–45, PPH was found in 24% of adults with obesity and 10% of the non-obesity group [24]. In a population-based study, the prevalence of PPH in individuals over 18 years old was reported to be 19.9% after a 75 g OGTT. In the same study, the prevalence of PPH increased with age, with rates of 12.5% in the 19–39 age group, 21.4% in the 40–49 age group, and 27.6% in those aged 70 and above [25]. Considering that the mean age of the participants in our study was 42, the PPH rate observed aligns with these findings. The results indicate that PPH can be a significant health issue not only in older adults but also in younger adults. Although postprandial hypoglycemia is often considered the first diagnosis in patients with postprandial symptoms, the likelihood of detecting PPH appears to be higher.

Symptoms such as fatigue, trembling, anxiety, palpitations, sweating, hunger, dizziness, blurred vision, and confusion occurring 2–5 h after meals often suggest postprandial hypoglycemia. However, in many patients with these complaints, biochemical evidence of hypoglycemia is not consistently found. In a study by Palardy et al. [13] involving 28 patients with suspected postprandial hypoglycemia, blood glucose levels measured during symptomatic episodes were below 50 mg/dL in only 5 patients (18%) and between 50 and 60 mg/dL in 8 (29%) patients. Similarly, another study of 26 patients with postprandial hypoglycemia symptoms found that although 10 patients exhibited symptomatic hypoglycemia during an OGTT, none of these patients developed symptomatic hypoglycemia when tested with an MMT [14]. This pattern was echoed in a study by Hogan [15], where 33 patients with suspected reactive hypoglycemia underwent an MMT, and none experienced blood glucose levels below 50 mg/dL. More recently, Hall [16] evaluated 40 patients with suspected reactive hypoglycemia and found that while 22 patients experienced symptoms during an OGTT, blood glucose levels fell below 55 mg/dL in only 12 patients (30%). Notably, none of the patients had their blood glucose level drop below 55 mg/dL during the MMT. In our study, despite 81.1% of patients reporting adrenergic symptoms and 34.2% reporting neuroglycopenic symptoms, only 10.8% had their blood glucose levels drop below 55 mg/dL during the MMT, consistent with findings in the literature.

Common symptoms of PPH include dizziness, a feeling of faintness, and blurred vision [21]. In elderly patients, PPH can lead to more serious consequences, such as falls, syncope, stroke, acute cardiovascular events, and even death. In a study by Asensio et al. [26], elderly individuals with PPH were found to experience neuroglycopenic symptoms such as fatigue and drowsiness, especially after breakfast and lunch. Another study observed symptoms such as chest pain, drowsiness, dizziness, and fatigue in elderly patients with PPH [27]. In our study, during the MMT, 30.4% of those with PPH exhibited adrenergic symptoms, while 47.8% experienced neuroglycopenic symptoms. Although these rates were higher than in patients without PPH, the difference did not reach statistical significance.

In this study, consistent with the literature, sBP and dBP levels at the start of the MMT were higher in individuals with PPH compared to those without. Similarly, in a study comparing 27 patients with PPH and 27 without PPH, pre-meal blood pressure was found to be significantly higher in those with PPH [11]. Other studies have also supported that higher sBP in hypertensive patients is a risk factor for PPH [28,29]. In our study, patients with known hypertension were excluded. Therefore, our results suggest that even in individuals without hypertension, elevated baseline blood pressure may be a risk factor for PPH.

Although various mechanisms have been proposed regarding the pathophysiology of PPH, its exact cause remains unclear. In simple terms, inadequate cardiovascular compensation in response to splanchnic blood pooling after a meal is suspected. Numerous factors, such as autonomic and neural dysfunction, changes in gastrointestinal hormones, meal composition, gastric distension, and the rate of nutrient transit to the small intestine, are suggested to play a role [30]. Many gastrointestinal hormones have been associated with PPH, including insulin, GLP-1, GLP-2, glucose-dependent insulinotropic polypeptide (GIP), somatostatin, calcitonin gene-related peptide (CGRP), neurotensin, and vasoactive intestinal peptide (VIP) [12,21].

Studies examining the relationship between PPH and insulin have yielded conflicting results. While some studies report that insulin has no effect on the development of PPH [31,32,33], others have concluded that an increase in insulin is associated with PPH [34,35]. The observation that PPH develops after oral glucose intake but not after fructose or xylose intake, which do not significantly stimulate insulin secretion, has been presented as indirect evidence of the relationship between insulin and PPH [36,37]. In a study by Hu et al. [11], it was demonstrated that the postprandial rise in blood glucose and insulin levels was significantly higher in patients with PPH compared to the control group. The relationship between PPH and insulin may be explained by insulin’s effects on vascular tone, vasodilation, and the autonomic nervous system [38]. Insulin increases nitric oxide release after meals, which dilates blood vessels and lowers blood pressure. In this process, peripheral vascular resistance decreases, potentially leading to a drop in blood pressure in the postprandial period [39]. In older subjects, nitric oxide mechanisms were found to mediate the fall in blood pressure, increase in heart rate, and decrease in insulin secretion after oral glucose intake, independent of gastric emptying or changes in incretin hormones [40]. It has been noted that these processes are more pronounced in individuals with autonomic nervous system dysfunction, which may cause a more severe drop in postprandial blood pressure [41].

Contrary to studies supporting the relationship between PPH and insulin, a study involving 24 patients with multisystem atrophy, a neurodegenerative disorder, found that postprandial glucose and insulin levels were similar between those with and without PPH, while the average increase in GLP-1 was greater in those with PPH [32]. In another study, 20 patients aged over 65 were administered intraduodenal infusions of saline (0.9%) or glucose at doses of 1, 2, or 3 kcal/min on different days for 60 min While increasing glucose doses led to higher glucose and insulin levels, the 1 kcal/min intraduodenal glucose infusion had no effect on blood pressure [33]. In our study, no relationship was found between blood pressure and insulin levels during the MMT in patients with PPH.

The prevalence of PPH has been reported to be high in people living with diabetes. In a study by Kitae et al. [5], PPH was observed in 50% of 300 people living with diabetes. Similarly, another study suggested that PPH in people living with diabetes might be associated with fluctuations in blood glucose levels [42]. However, the relationship between glucose, PPH, and diabetes remains unclear. In one study conducted on older adults without diabetes, those with PPH exhibited a more pronounced drop in sBP and dBP in response to oral glucose compared to those without PPH [43]. Moreover, in another study, 8 healthy elderly individuals were administered intraduodenal glucose at a rate of 1 or 3 kcal/min for 60 min, and it was reported that only the 3 kcal/min glucose infusion caused a significant drop in sBP and dBP along with an increase in heart rate [44]. In a study including elderly individuals, a relationship was found between the maximum postprandial decrease in sBP and maximum increase in insulin, while no association was observed with maximum glucose increase [11]. In our study, similar with insulin, no relationship was found between PPH and glucose. It should be emphasized that, unlike many previous studies examining the relationship between PPH and insulin or glucose, our study focused on younger adults, and we excluded individuals with additional conditions that could be risk factors for PPH, such as neurodegenerative diseases, DM, hypertension, and rheumatic diseases. Additionally, in contrast to most prior research, we used an MMT, which can be suggested to better reflect real-life conditions.

One of the limitations of our study is the lack of measurement of gastrointestinal hormones, such as glucagon, GLP-1, GLP-2, GIP, somatostatin, and VIP, and hormones indicating sympathetic nervous system response, such as norepinephrine, which may play roles in the pathogenesis of PPH. However, as the primary aim of our study was to investigate the role of insulin and glucose in the pathogenesis of PPH, these parameters were not measured. Although a larger sample size was included compared to many studies in the literature, it can be said that the number of PPH patients may still be insufficient to draw definitive conclusions. Another limitation of our study is the absence of measurement of gastric emptying and autonomic nerve function tests, both of which may contribute to the symptoms of PPH. Nonetheless, this is the first study to assess the prevalence of PPH in a healthy adult population with postprandial symptoms.

## 5. Conclusions

PPH may be responsible for the symptoms in a significant proportion of adults with postprandial symptoms. This condition appears to be more common than postprandial hypoglycemia. The lack of a relationship between PPH and insulin or glucose suggests that other hormones, such as incretins and norepinephrine, may play roles in the pathogenesis of PPH in healthy adults. Further studies are needed in this field to better understand the mechanisms underlying PPH, especially in healthy adults.

## Figures and Tables

**Table 1:** Demographic and clinical features and basal laboratory results of patients.

	Median [IQR]/*n* (%) *n* = 111
Age (years)	42 [17]
Sex (female/male)	86 (77.5)/25 (22.5)
Body weight (kg)	67.0 [14.0]
Body mass index (kg/m^2^)	24.8 [4.6]
Adrenergic symptoms	90 (81.1)
Neuroglycopenic symptoms	38 (34.2)
Fasting blood glucose (mg/dL) (*n* = 108)	82.0 [12.8]
Fasting insulin (mU/L) (*n* = 84)	7.6 [5.1]
Fasting C-peptide (µg/L) (*n* = 53)	1.4 [1.1]
Postprandial blood glucose (mg/dL) (*n* = 76)	80.0 [25.5]
Postprandial insulin (mU/L) (*n* = 18)	21.7 [30.5]
HbA1c (%) (*n* = 99)	5.4 [0.4]
ACTH (pg/mL) (*n* = 62)	20.7 [14.7]
Cortisol (µg/dL) (*n* = 63)	14.6 [6.1]
Patients with PPH during MMT	26 (23.4)
Patients with postprandial hypoglycemia during MMT	12 (10.8)

HbA1c: Hemoglobin A1c, ACTH: Adrenocorticotropic hormone, PPH: Postprandial hypotension, MMT: Mixed meal test.

**Table 2:** Demographic and clinical features of patients with and without postprandial hypotension after exclusion of people with postprandial hypoglycemia.

	PPH (*n* = 23)	Without PPH (*n* = 76)	*p*
Age (years)	40 [13]	41 [19]	0.546
Female/Male	16 (69.6)/14 (18.4)	62 (81.6)/7 (30.4)	0.249
Adrenergic symptoms	17 (73.9)	63 (82.9)	0.338
Neuroglycopenic symptoms	8 (34.8)	28 (36.8)	0.857
HbA1c (%) (*n* = 21/66)	5.4 [0.5]	5.4 [0.4]	0.586
ACTH (pg/mL) (*n* = 12/43)	20.6 [11.8]	22.0 [19.9]	0.984
Cortisol (µg/dL) (*n* = 12/43)	13.4 [3.2]	15.6 [6.3]	0.014
Adrenergic symptoms during MMT	7 (30.4)	13 (17.1)	0.234
Neuroglycopenic symptoms during MMT	11 (47.8)	22 (28.9)	0.129
Glucose at the start of MMT (mg/dL)	79.0 [9.0]	80.5 [11.5]	0.518
Insulin at the start of MMT (mU/L)	6.7 [3.5]	6.6 [5.6]	0.737
C-peptide at the start of MMT (µg/L)	1.5 [0.8]	1.4 [0.7]	0.964
sBP at the start of MMT (mmHg)	123 [30]	111 [17]	0.002
dBP at the start of MMT (mmHg)	80 [16]	74 [11]	0.010
Heart rate at the start of MMT (bpm)	80 [15]	80 [14]	0.388
Lowest glucose during MMT (mg/dL)	70.0 [11.0]	71.0 [12.0]	0.605
Highest insulin during MMT (mU/L)	68.9 [52.4]	66.0 [48.6]	0.957
Highest C-peptide during MMT (µg/L)	5.0 [3.29]	5.1 [3.24]	0.911
Maximum fall in sBP (mmHg)	24 [8]	10 [9]	<0.001
Maximum rise in heart rate (bpm)	9 [7]	7 [8]	0.606

HbA1c: Hemoglobin A1c, ACTH: Adrenocorticotropic hormone, PPH: Postprandial hypotension, sBP: Systolic blood pressure, dBP: Diastolic blood pressure, MMT: Mixed meal test.

**Table 3:** Correlation between glucose, insulin, and C-peptide with systolic blood pressure at all times in people with and without postprandial hypotension after exclusion of people with postprandial hypoglycemia.

		sBP (mmHg)							
		0 min	30 min	60 min	90 min	120 min	180 min	240 min	300 min
Glucose (mg/dL)	r	−0.011	0.270	0.419	0.407	0.194	0.181	−0.037	0.003
	*p*	0.912	0.007	<0.001	<0.001	0.055	0.073	0.718	0.975
Insulin (mU/L)	r	−0.064	−0.042	0.298	0.344	0.197	0.333	0.220	0.142
	*p*	0.531	0.679	0.003	<0.001	0.050	0.001	0.029	0.160
C-peptide (µg/L)	r	0.230	0.076	0.263	0.373	0.333	0.424	0.315	0.283
	*p*	0.022	0.457	0.008	<0.001	0.001	<0.001	0.001	0.005

sBP: Systolic blood pressure.

**Table 4:** Correlation between glucose, insulin, and C-peptide with systolic blood pressure at all times in people with postprandial hypotension after exclusion of people with postprandial hypoglycemia.

		sBP (mmHg)							
		0 min	30 min	60 min	90 min	120 min	180 min	240 min	300 min
Glucose (mg/dL)	r	0.218	−0.026	0.330	0.490	0.313	0.164	−0.025	−0.035
	*p*	0.319	0.906	0.124	0.018	0.146	0.454	0.910	0.873
Insulin (mU/L)	r	−0.193	−0.319	0.122	0.216	0.277	0.318	0.081	0.232
	*p*	0.378	0.138	0.579	0.322	0.201	0.140	0.713	0.287
C-peptide (µg/L)	r	0.278	−0.184	0.092	0.326	0.214	0.285	0.184	0.324
	*p*	0.199	0.399	0.677	0.129	0.326	0.187	0.402	0.131

sBP: Systolic blood pressure.

**Table 5:** Correlation between glucose, insulin, and C-peptide with the magnitude of systolic blood pressure fall in individuals with and without postprandial hypotension, excluding postprandial hypoglycemia cases.

sBP (mmHg)								
		Δ0–30 min	Δ30–60 min	Δ60–90 min	Δ90–120 min	Δ120–180 min	Δ180–240 min	Δ240–300 min
Glucose (mg/dL)	r	−0.020	−0.312	0.015	0.154	−0.067	−0.007	−0.061
	*p*	0.848	0.002	0.884	0.128	0.509	0.942	0.550
Insulin (mU/L)	r	0.108	−0.241	−0.025	0.174	−0.124	0.011	0.149
	*p*	0.288	0.016	0.804	0.085	0.222	0.917	0.140
C-peptide (µg/L)	r	0.035	−0.221	0.071	0.028	−0.057	0.068	0.028
	*p*	0.729	0.028	0.483	0.780	0.575	0.506	0.781

sBP: Systolic blood pressure.

**Table 6:** Correlation between glucose, insulin, and C-peptide with the magnitude of systolic blood pressure fall in individuals with postprandial hypotension, excluding postprandial hypoglycemia cases.

sBP (mmHg)								
		Δ0–30 min	Δ30–60 min	Δ60–90 min	Δ90–120 min	Δ120–180 min	Δ180–240 min	Δ240–300 min
Glucose (mg/dL)	r	0.108	−0.194	0.024	0.191	0.032	0.064	0.071
	*p*	0.625	0.375	0.914	0.382	0.883	0.773	0.748
Insulin (mU/L)	r	0.262	−0.420	0.019	−0.142	−0.057	0.177	−0.125
	*p*	0.227	0.046	0.932	0.519	0.798	0.420	0.570
C-peptide (µg/L)	r	0.300	−0.564	−0.006	0.021	−0.003	0.103	−0.097
	*p*	0.164	0.005	0.977	0.923	0.989	0.640	0.659

sBP: Systolic blood pressure.

## Data Availability

The data in this article will be shared upon reasonable request.

## Abbreviations

PPH	Postprandial hypotension
MMT	Mixed meal test
sBP	Systolic blood pressure
dBP	Diastolic blood pressure
DM	Diabetes mellitus
GLP	Glucagon-like peptide
OGTT	Oral glucose tolerance test
HbA1c	Hemoglobin A1c
ACTH	Adrenocorticotropic hormone
GIP	Glucose-dependent insulinotropic polypeptide
CGRP	Calcitonin gene-related peptide
VIP	Vasoactive intestinal peptide
ECG	Electrocardiogram
